# A Phase-Based, Multidisciplinary Enhanced Recovery Pathway for Bariatric Procedures: The EUropean PErioperative MEdical Networking (EUPEMEN) Collaborative for Obesity Surgery

**DOI:** 10.3390/jcm15051706

**Published:** 2026-02-24

**Authors:** Orestis Ioannidis, Elissavet Anestiadou, Jose M. Ramirez, Nicolò Fabbri, Javier Martínez Ubieto, Carlo Vittorio Feo, Antonio Pesce, Kristyna Rosetzka, Antonio Arroyo, Petr Kocián, Luis Sánchez-Guillén, Ana Pascual Bellosta, Adam Whitley, Alejandro Bona Enguita, Marta Teresa-Fernandéz, Stefanos Bitsianis, Savvas Symeonidis

**Affiliations:** 1 Fourth Department of Surgery, Medical School, Faculty of Health Sciences, Aristotle University of Thessaloniki, General Hospital “George Papanikolaou”, 57010 Thessaloniki, Greece; elissavetxatz@gmail.com (E.A.); sbitsiani@gmail.com (S.B.); simeonidissavvas@yahoo.com (S.S.); 2 Institute for Health Research Aragón, 50009 Zaragoza, Spain; jramirez@unizar.es (J.M.R.); jmtezubieto@hotmail.com (J.M.U.); anapascual689@gmail.com (A.P.B.); secretariagerm@gmail.com (A.B.E.); mteresa@iisaragon.es (M.T.-F.); 3 Department of Surgery, Faculty of Medicine, University of Zaragoza, 50009 Zaragoza, Spain; 4 Department of Surgery, Azienda Unità Sanitaria Locale Ferrara—University of Ferrara, 44121 Ferrara, Italy; n.fabbri@ausl.fe.it (N.F.); cvfeo@unife.it (C.V.F.); antonio.pesce@ausl.fe.it (A.P.); 5 Department of Anesthesia, Resuscitation and Pain Therapy, Miguel Servet University Hospital, 50009 Zaragoza, Spain; 6 Department of Plastic Surgery, Second Faculty of Medicine, Charles University and Motol University Hospital, 150 06 Prague, Czech Republic; kristina.rosetzua@gmail.com; 7 Department of Surgery, Universidad Miguel Hernández Elche, Hospital General Universitario Elche, 03203 Elche, Spain; arroyocir@hotmail.com (A.A.); drsanchezguillen@gmail.com (L.S.-G.); 8 Grupo Español de Rehabilitación Multimodal (GERM), 50009 Zaragoza, Spain; 9 Department of Surgery, Second Faculty of Medicine, Charles University and Motol University Hospital, 150 06 Prague, Czech Republic; kocian.cz@gmail.com; 10 Department of Surgery, University Hospital Kralovske Vinohrady, 100 34 Prague, Czech Republic

**Keywords:** bariatric surgery, metabolic surgery, Enhanced Recovery After Surgery, ERAS, EUPEMEN protocol, multidisciplinary perioperative care, prehabilitation

## Abstract

**Background/Objectives**: Obesity remains a major global health burden, with metabolic–bariatric surgery being the most efficient long-term treatment strategy. However, both variability in perioperative care and postoperative complications persist. To address these challenges, the EUropean PErioperative MEdical Networking (EUPEMEN) protocol for bariatric surgery was developed to standardize care and enhance perioperative outcomes across European healthcare settings. **Methods**: The protocol was formulated through close collaboration among experts from multiple disciplines, involving surgeons, anesthetists, nurses, and nutritionists. Its development included a literature review, expert consensus, and the creation of structured perioperative guidelines covering the preoperative, intraoperative, and postoperative phases. Focus areas include patient education, nutritional optimization, early mobilization, opioid-sparing analgesia, and minimally invasive surgical techniques, supported by educational materials and manuals. Technical activities included the development of detailed multimodal rehabilitation manuals translated into five languages, the creation of an open-access online learning platform, training of future educators through a “train the trainer” approach, organization of multiplier promotional events, international collaboration meetings to refine the protocol, and revision and standardization of existing perioperative care guidelines to ensure evidence-based, unified practices across Europe. **Results**: Implementation of the EUPEMEN protocol aims to reduce postoperative complications, enhance recovery, and decrease hospitalization time. Standardized rehabilitation pathways and access to free educational platforms promote consistent care delivery across diverse healthcare environments. Key strategies include early oral intake, limited use of invasive devices, and comprehensive patient preparation. **Conclusions**: The EUPEMEN protocol introduces an evidence-based, multidisciplinary framework for optimizing perioperative management in bariatric surgery. While variability in resources and adherence may present potential obstacles, its application holds significant promise for improving perioperative outcomes. Future studies are necessary to assess its long-term impact and adaptability in different healthcare settings.

## 1. Introduction

The obesity pandemic constitutes one of the most critical public health issues of the 21st century worldwide, with a continuously increasing global prevalence and a well-described association with cardiometabolic pathologies, including cardiovascular disease, type 2 diabetes mellitus, as well as obstructive sleep apnea (OSA), musculoskeletal degeneration, malignancy, and premature mortality [1]. Notably, the obesity epidemic is no longer limited to high-income countries, increasingly affecting low- to middle-income regions, predominantly within urban populations, where the rate of overweight and obese children and adolescents continues to rise alarmingly [2]. Nowadays, evidence-based management of obesity is multifaceted and encompasses five principal therapeutic strategies: behavioral modification, nutritional intervention, structured physical activity, pharmacotherapy, and metabolic–bariatric procedures—with comprehensive care plans incorporating selected strategies according to individual patient characteristics and disease severity [3].

Metabolic–bariatric surgery (MBS) has emerged as the most effective long-term management option for severe obesity, offering long-term weight reduction with favorable metabolic outcomes, when compared with lifestyle or pharmacological interventions [4]. Indeed, emerging evidence highlight that MBS is associated with increased and more durable weight loss as well as lower long-term costs compared with GLP-1 receptor agonists in class II (body mass index [BMI] ≥ 35) and class III (BMI ≥ 40) obese patients, challenging the traditional perception of MBS as a last-resort therapy and underscoring the crucial need to reconsider its position within contemporary obesity treatment algorithms [5]. Nevertheless, bariatric patients are characterized a complex perioperative profile, characterized by multimorbidity, altered respiratory mechanics, increased thromboembolic risk, insulin resistance, and a high prevalence of micronutrient deficiencies. These features make the perioperative management of bariatric patients particularly demanding and necessitate a structured, multidisciplinary approach [6,7].

Firstly described in 1997 by Henrik Kehlet, Enhanced Recovery After Surgery (ERAS) principles were introduced to limit the perioperative stress response, preserve organ function, and accelerate patient recovery after surgery through evidence-based multimodal perioperative care [8,9]. The first evidence-based guidelines specifically addressing perioperative care in MBS were developed by the Enhanced Recovery After Surgery (ERAS) Society in 2016, as a response to the rapidly increasing global volume of bariatric procedures and the lack of consensus regarding optimal perioperative management [10]. Notably, a large number of recommendations were extracted from non-bariatric protocols, principally from colorectal procedures [10]. The comprehensive, updated evidence-based version of the ERAS consensus, published in 2022, further expanded this framework; however, the quality of evidence for several ERAS suggestions remains limited in the bariatric setting, often leading to extrapolation from data derived from other surgical settings [11]. Despite strong research evidence supporting the feasibility and importance of ERAS pathways in MBS, their implementation in daily clinical practice remains inconsistent across institutions, mainly due to resistance to change, organizational and resource barriers, limited multidisciplinary coordination, and lack of structured educational frameworks [12]. Furthermore, bariatric patients exhibit a set of unique physiological and behavioral characteristics, including altered pharmacokinetics, high rates of postoperative nausea and vomiting (PONV), frequent OSA, and the need for lifelong nutritional surveillance, which further limit the applicability of generic ERAS pathways without tailored adaptation [13].

The EUropean PErioperative MEdical Networking (EUPEMEN) project has emerged as one of the most structured collaborative efforts in Europe to bridge the gap between perioperative theoretical science and everyday clinical practice. This project clearly stated that the primary challenge in perioperative medicine is not the generation of new evidence, but the difficulty of integrating existing knowledge into routine care. The EUPEMEN initiative established a pan-European, phase-based framework to harmonize perioperative care through standardized multimodal rehabilitation manuals, multidisciplinary role allocation, and structured educational dissemination, emphasizing that sustainable ERAS implementation requires continuous auditing, team engagement, and adaptation to local clinical contexts [14,15,16,17,18,19]. 

The primary aim of this study is to present a structured, phase-based enhanced recovery protocol specifically designed for MBS, developed within the EUPEMEN collaborative framework. The protocol is intended to serve as a standardized perioperative care pathway adaptable across different healthcare systems. It is based on integration of current scientific evidence, structured literature review, and multidisciplinary expert consensus using a Delphi-based methodology. Therefore, this work should be interpreted as an evidence-informed, consensus-driven protocol proposal rather than solely a narrative summary of existing practices. In addition, the protocol aims to facilitate standardized perioperative data collection, multidisciplinary coordination, and reproducible implementation of enhanced recovery principles in real-world clinical settings.

## 2. Materials and Methods

### 2.1. Protocol and Consensus Formation

The EUPEMEN pathway was created through collaboration among five academic tertiary referral centers across four European countries (Spain, Italy, the Czech Republic, and Greece). Participating institutions included Fundación Instituto de Investigación Sanitaria Aragón and Universidad Miguel Hernández de Elche (Spain), Azienda Unità Sanitaria Locale Ferrara–University of Ferrara (Italy), Charles University and Motol University Hospital (Czech Republic), and Papanikolaou General Hospital of Thessaloniki (Greece) [16].

Each center contributed theoretical knowledge and professional competence from surgery, anesthesiology, nutrition, physiotherapy, and perioperative nursing experts.

These institutions contribute clinical experience in perioperative surgical management and participate in research initiatives focused on evidence-based perioperative care and recovery optimization. The collaboration aimed to integrate clinical expertise with implementation-oriented research experience in order to develop a structured, clinically applicable perioperative rehabilitation protocol.

Participating experts were selected based on documented clinical and academic expertise in surgical and perioperative management of adult patients. Selection criteria included active clinical involvement in bariatric or perioperative surgical care programs, participation in academic or high-volume clinical centers, and demonstrated scientific contribution through peer-reviewed publications, research collaboration, or involvement in guideline, consensus, or protocol development initiatives. The selection process emphasized multidisciplinary clinical experience, research engagement, and implementation expertise rather than strict numerical thresholds.

The protocol was developed with focus on adult surgical populations, including patients with complex medical histories and heterogeneous physiological responses to perioperative treatment. In this context, the protocol development team consisted primarily of surgeons and perioperative specialists with clinical experience in adult surgical care pathways. Where applicable, participating centers were also characterized by established bariatric surgical activity and structured perioperative care pathways.

A structured three-round Delphi procedure involving surgeons, anesthetists, dietitians, physiotherapists, and specialized nurses from the participating centers was followed to achieve consensus [20]. Recommendations were distributed anonymously, revised iteratively following feedback, and received their final form during online consensus meetings. Statements were incorporated into the EUPEMEN protocol only when at least 75% agreement was reached among participants. A final external review phase was conducted to evaluate feasibility and applicability of the suggestions in routine clinical practice. 

In parallel, a structured literature review was performed to identify evidence-based perioperative interventions relevant to MBS and findings from this search were used to update the Delphi process, ensuring that all consensus statements are grounded in the most current clinical evidence. A focused literature search was conducted in PubMed, Scopus, and Cochrane Library to retrieve studies addressing perioperative care in MBS. Search terms included combinations of “bariatric surgery”, “enhanced recovery”, “perioperative management”, and “metabolic surgery”, and only peer-reviewed publications in English from the preceding 15 years were considered. The selection process strictly followed the PRISMA methodology, and the strength of evidence supporting individual interventions was assessed according to the GRADE framework, giving priority to randomized controlled trials, systematic reviews, and robust cohort studies. Additionally, the existing RICA (Recovery Intensification for Optimal Care in Adult Surgery) framework was reviewed and adapted to reflect the contemporary evidence and the specific perioperative requirements of bariatric patients [21]. This harmonization process ensured consistency between suggestions of the EUPEMEN bariatric protocol and previously validated enhanced recovery pathways, facilitating standardized implementation across participating healthcare facilities. The revised RICA-based structure was subsequently incorporated into the final consensus recommendations.

### 2.2. Technical Activities, Implementation Tools and Dissemination of the Protocol

To promote real-world clinical adoption of the project, a series of technical activities accompanied protocol development. These included the preparation of multimodal rehabilitation manuals, providing structured, step-by-step instructions on perioperative care for MBS and additional surgical subspecialties, including esophageal, colorectal, liver, and gastric surgery, translation of all materials into English, Spanish, Italian, Greek, and Czech, and their dissemination via the EUPEMEN open-access digital platform. An online educational environment was created to host the protocols and training modules, supported by a train-the-trainer strategy to enhance sustainability. Finally, regular international meetings and workshops were held, and the existing RICA (Recovery Intensification for Optimal Care in Adult Surgery) pathway was updated, incorporating current evidence [21].

From a logistical perspective, protocol implementation is supported through standardized multimodal rehabilitation manuals, multilingual educational materials, digital learning platforms, and structured training programs based on a train-the-trainer model. These tools are designed to facilitate reproducible implementation across healthcare systems with different organizational structures while maintaining adherence to evidence-based perioperative principles.

In addition to improving patient care and perioperative outcomes, the EUPEMEN protocol supports healthcare professionals through structured perioperative data collection. The phase-based design enables standardized and detailed documentation of preoperative risk factors, intraoperative parameters, and postoperative recovery indicators. In addition, this structured data collection facilitates continuous clinical monitoring, supports early detection of deviations from expected recovery pathways, and enhances multidisciplinary decision-making. Furthermore, standardized datasets support quality improvement initiatives, institutional auditing processes, and future research development.

From an ethical standpoint, the EUPEMEN protocol does not introduce experimental interventions but represents a structured implementation of evidence-based perioperative care derived from published literature, international guidelines, and expert consensus. Therefore, it is applied within standard clinical care pathways and complies with institutional clinical governance policies. When data are collected for audit or research purposes, they are anonymized and processed according to local data protection regulations and international ethical standards. 

## 3. The EUPEMEN Protocol in Metabolic–Bariatric Surgery

The finalized bariatric protocol is organized into three sequential phases: pre-admission, perioperative (covering intraoperative and immediate postoperative care), and postoperative management until discharge. For each phase, specific evidence-based actions are defined, combined with clear allocation of responsibilities among surgeons, anesthetists, nurses, dietitians, physiotherapists, and allied professionals. The EUPEMEN Protocol for Bariatric Surgery is presented in detail in the Supplementary Materials (The EUPEMEN Protocol). 

The protocol was primarily designed for adult patients undergoing elective primary metabolic and bariatric surgical procedures. The phase-based structure allows adaptation to higher-risk populations, including patients with super-obesity (BMI ≥ 50 kg/m^2^), revisional bariatric surgery, and patients with complex comorbidity profiles, based on institutional experience and individualized perioperative risk assessment. Pediatric bariatric surgery was not specifically addressed during protocol development and therefore falls outside the primary intended scope of the current protocol.

The protocol was developed using perioperative care principles derived primarily from the evidence base of the most frequently performed bariatric procedures, including laparoscopic sleeve gastrectomy and Roux-en-Y gastric bypass. However, the EUPEMEN framework is structured as a phase-based perioperative pathway rather than a procedure-specific protocol. As such, most components are intended to be universally applicable across bariatric operations, including one-anastomosis gastric bypass and other metabolic procedures, while allowing procedure-specific adaptations according to surgical complexity, anatomical considerations, and institutional expertise.

**Figure 1:**
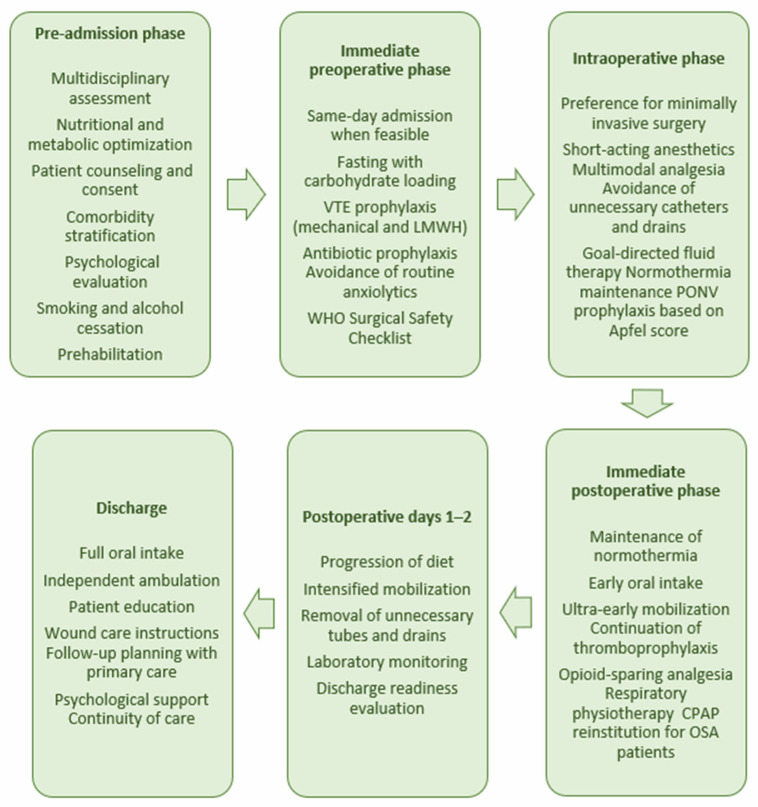
Schematic presentation of the EUPEMEN enhanced recovery pathway for metabolic–bariatric surgery, structured into six consecutive phases: pre-admission, immediate preoperative, intraoperative, immediate postoperative, postoperative days 1–2, and discharge. Each phase summarizes the key multidisciplinary interventions designed to optimize perioperative care, promote early functional recovery, reduce postoperative complications, and facilitate safe discharge and continuity of care.

The structure and content of the EUPEMEN bariatric enhanced recovery pathway are presented in Figure 1.

### 3.1. Pre-Admission Phase

The preoperative phase offers a critical time window for the optimization of patients undergoing MBS and represents a cornerstone of enhanced recovery pathways. This phase is based on the coordinated participation of surgeons, anesthetists, nurses, dietitians, and allied health professionals, in accordance with the multidisciplinary, phase-based structure promoted by the EUPEMEN framework [19,22,23]. Comprehensive patient counseling should be provided in both verbal and written form and should be optimally conducted by a multidisciplinary care team comprising the bariatric surgeon, registered dietitian, a behavioral medicine specialist, nurse educators, and, when indicated, subspecialty providers such as cardiologists, pulmonologists, endocrinologists, and anesthesiologists, outlining the surgical procedure, expected perioperative course, discharge criteria, early mobilization goals, dietary progression, and the long-term lifestyle commitments required after surgery. As part of this structured preoperative process, written informed consent must be obtained after comprehensive discussion with the patient. This comprehensive, team-based approach ensures that surgical candidacy is appropriately assessed, perioperative risks are minimized, and patient expectations and adherence to postoperative lifestyle changes are adequately addressed. Preoperative patient education improves adherence, reduces perioperative anxiety and has been associated with improved postoperative outcomes within ERAS programs [24]. In parallel, all MBS candidates should undergo a thorough medical evaluation including full medical and surgical history, physical examination, electrocardiography, and targeted imaging or laboratory investigations upon, including a baseline of coagulation parameters, biochemical profile, nutritional profile and full blood count. Particular attention should be given on cardiovascular and respiratory disorders, and metabolic comorbidities. Preoperative spirometry is recommended in patients with suspected or established restrictive lung disease [25,26]. Given the high rates of OSA among bariatric patients, structured screening using preoperative risk stratification validated tools, such as the STOP-BANG test, is mandatory and patients with score with a score ≥ 3 should be referred for further evaluation, such as polysomnography, if perioperative continuous positive airway pressure planning [27,28]. In addition, a formal cardiology assessment is suggested in patients presenting with more than three cardiovascular risk factors, as well as for all individuals with recent-onset or active cardiovascular disease before MBS [29]. Last but not least, preoperative workup should also include upper gastrointestinal endoscopy, including systematic evaluation of the esophagus, stomach, and duodenum, as well as screening for *Helicobacter pylori*, and eradication therapy must be completed prior to surgery in case of infection.

Diabetes mellitus and perioperative hyperglycemia, apart from their strong association with increased postoperative complications, higher in-hospital mortality rates, and increased overall hospitalization costs, are also important factors that guide selection of the most appropriate MBS technique [30]. Evidence from randomized trials indicates that procedures such as mini-gastric bypass, biliopancreatic diversion, Roux-en-Y gastric bypass, and sleeve gastrectomy lead to greater diabetes remission rates than conservative treatment, underscoring the importance of incorporating diabetic status into preoperative surgical planning [31]. Accordingly, preoperative assessment of fasting blood glucose and glycated hemoglobin is essential before MBS, and poorly controlled diabetes must prompt referral for metabolic optimization, as perioperative glycemic variability has been linked to adverse postoperative outcomes [32]. Preoperative nutritional optimization is fundamental to enhanced recovery in MBS, contributing to risk reduction and improved postoperative outcomes. Implementation of low-calorie diets or commercial weight-loss formulas facilitates preoperative weight reduction and decreases hepatic parenchyma volume, while adjunctive strategies such as pharmacotherapy or intragastric balloon placement may be applied in selected patients [33]. Concurrently, systematic assessment and correction of nutritional deficiencies, particularly calcium, iron, vitamin D, and vitamin B12 with additional testing (e.g., thiamine, zinc, selenium) considered in high-risk patients or those with suspected malnutrition, are mandatory prior to surgery to minimize perioperative complications and support long-term metabolic health in collaboration with specialized dietetic support. Any deficiencies should be corrected prior to surgery in line with ESPEN clinical nutrition guidelines [30]. Preoperative correction of anemia and iron deficiency is particularly important, since perioperative anemia has been correlated with adverse perioperative outcomes [34].

Preoperative weight optimization strategies should be individualized according to patient risk profile, metabolic status, and surgical complexity [35]. Short-term preoperative very-low-calorie diets (VLCDs) are commonly recommended for approximately 2–4 weeks prior to surgery, particularly in patients with severe obesity, central adiposity, metabolic syndrome, or evidence of hepatic steatosis, where liver volume reduction may facilitate safer surgical exposure [36]. Nutritional optimization should also include systematic screening for micronutrient deficiencies, such as iron, vitamin B12, vitamin D, folate, evaluation of protein intake, and structured dietitian-guided nutritional planning [37]. In selected high-risk patients, longer preoperative nutritional optimization periods may be considered based on clinical judgment and institutional protocols. Patient selection for intensive preoperative weight reduction strategies should consider surgical risk, metabolic control, and anticipated technical complexity [38].

Adequate protein intake represents a key component of preoperative nutritional optimization, particularly in patients at risk of sarcopenia or protein malnutrition. In most bariatric candidates, a daily protein intake of approximately 1.0–1.5 g/kg of ideal body weight is generally targeted, adjusted according to nutritional status, renal function, and metabolic profile [39]. In patients undergoing preoperative caloric restriction, preservation of lean body mass should be prioritized through adequate protein supplementation and dietitian-guided nutritional planning. The routine use of preoperative immunonutrition in bariatric surgery remains controversial. While immunonutrition formulas enriched with arginine, omega-3 fatty acids, and nucleotides have demonstrated benefits in selected gastrointestinal surgical populations, current evidence in bariatric surgery remains limited [40]. Therefore, immunonutrition may be considered selectively in high-risk patients with malnutrition, chronic inflammation, or complex revisional procedures, based on multidisciplinary assessment [41]. Preoperative assessment of body composition and sarcopenia risk may provide additional prognostic information beyond body mass index alone. When available, screening tools such as bioelectrical impedance analysis, CT-based muscle mass assessment, or functional measures including handgrip strength and gait speed may help identify patients at increased risk of postoperative complications and delayed recovery [42]. In such patients, targeted nutritional and physical prehabilitation strategies should be considered. All preoperative nutritional strategies should be individualized and delivered within a multidisciplinary perioperative care framework.

A structured preoperative exercise program should be prescribed preoperatively, incorporating cardiovascular, respiratory, and muscle-strengthening activities tailored to the patient’s functional capacity. This targeted physical conditioning aims to enhance cardiorespiratory fitness, preserve lean body mass, and facilitate early postoperative mobilization. Psychological assessment with validated psychometric tools is also recommended to evaluate eligibility and readiness for surgery, behavioral patterns, and ability to adhere to long-term lifestyle modifications postoperatively [43]. ΒΜS candidates should be strongly encouraged to abandon tobacco use and significantly reduce alcohol consumption at least four weeks prior to surgery [44]. Finally, although the implementation of structured prehabilitation programs may be challenging in patients with obesity, such interventions, including physical exercises aiming at cardiovascular, respiratory and individualized muscle-strengthening exercises adapted to the patient’s physical condition, are likely to promote the establishment of regular and sustained physical activity patterns, thereby supporting functional recovery after MBS [45,46].

During the preadmission phase, the anesthetic risk should be documented using the American Society of Anesthesiologists (ASA) score, as recommended in bariatric ERAS guidelines [11]. Finally, the risk of PONV should be assessed using validated tools, allowing individualized prophylactic strategies to be planned during the perioperative period. Among them, the Apfel score is the basic tool used to assess the risk of PONV [47].

### 3.2. Perioperative Phase

#### 3.2.1. Immediate Preoperative Phase

The principal aim of the healthcare team during the immediate preoperative phase is to prepare the patient for MBS while minimizing perioperative stress and complications. Whenever feasible, admission should be scheduled on the day of the surgery to reduce unnecessary hospitalization and costs and improve patient flow. The anesthetist, the surgeon, and the nursing team hold a crucial role during coordination of this stage.

Under standard circumstances, patients are advised to follow a standardized fasting protocol, abstaining from solid food for at least 8 h before anesthesia induction while being permitted to consume clear liquids up to 2 h before surgery [48]. In the absence of contraindications, the administration of a single oral carbohydrate drink containing 12.5% maltodextrins (400 mL) is recommended approximately 2 h before surgery. Numerous literature reports highlight that preoperative carbohydrate solutions, when administered prior to elective surgery, have been shown to attenuate the development of insulin resistance postoperatively and improve indices of patient comfort, such as reduced sense of hunger and thirst, anxiety and nausea, compared with traditional fasting, without increasing the risk of aspiration, suggesting a potentially beneficial role in perioperative metabolic management within enhanced recovery protocols [49,50]. Under the same setting of ERAS protocols, routine anxiolytic premedication should be avoided in order to facilitate early postoperative mobilization and recovery. If hair removal is required at the incision site, it should be performed using an electric razor to limit the risk of surgical site infections.

Obesity is a well-recognized independent factor predisposing to venous thromboembolism, encompassing deep vein thrombosis and pulmonary embolism events, with the likelihood of thrombotic events rising in parallel with increased BMI and being especially pronounced in individuals with central adiposity [51]. Accordingly, mechanical thromboprophylaxis with compression stockings or intermittent pneumatic compression devices should be preoperatively applied based on the individual patient’s thromboembolic risk profile. In addition, pharmacological thromboprophylaxis with low-molecular-weight heparin should be introduced in the immediate preoperative period, ideally between 2 and 12 h preoperatively, with the timing tailored to the anticipated induction of neuraxial anesthesia [51]. 

Surgical interventions in obese patients are strongly associated with an increased rate of postoperative complications, such as surgical site infections, with reported rates after bariatric surgery approaching 15%. In this context, prophylactic antibiotics must be administered within 30–60 min before surgical incision, with the choice of regimen determined according to local institutional policy [52]. For patients with delayed gastric emptying or other risk factors for aspiration, appropriate prophylactic measures should be implemented to avoid gastric regurgitation during anesthesia induction. These may include the administration of prokinetic or acid-suppressive agents, careful airway management planning, and the use of rapid-sequence induction techniques when clinically indicated.

#### 3.2.2. Intraoperative Phase

The intraoperative phase of MBS operations is jointly coordinated by the anesthetist, surgeon, and nursing team and is of pivotal importance for the success of enhanced recovery pathway. Evidence indicates that adoption of the World Health Organization Surgical Safety Checklist is associated with improved perioperative outcomes and reduced complication rates, with negligible financial cost. However, its effectiveness is highly dependent on consistent and complete adherence, underscoring the importance of structured training and team engagement within the operating theatre [53,54]. Prior to incision, the World Health Organization Surgical Safety Checklist must be completed to ensure correct patient identification, verification of the planned procedure, availability of essential equipment, and anticipation of potential intraoperative risks.

During the procedure, the anesthetist controls patient homeostasis through routine and systematic monitoring, which includes monitoring of vital signs, fraction of inspired oxygen (FiO_2_), depth of anesthesia, neuromuscular blockade, and blood glucose levels, together with non-invasive hemodynamic monitoring whenever available. Invasive arterial catheterization is not routinely used and should be applied to patients with severe cardiorespiratory diseases or anticipated hemodynamic instability [55]. Likewise, routine central venous catheter placement should be avoided in the absence of specific indications such as major resections or increased risk of renal failure postoperatively. In candidates with severe or morbid obesity, central venous catheterization is technically challenging because surface anatomical landmarks are frequently obscured, increasing the risk of failed attempts and procedural complications. When central venous access is clinically indicated, ultrasound guidance should be employed to enhance success rates and minimize complications by allowing real-time visualization of vascular anatomy and needle and guidewire advancement [56]. Routine bladder catheterization is also discouraged to reduce urinary tract infection (UTI) risk and to facilitate early postoperative mobilization [57]. However, given that postoperative urinary retention may adversely affect outcomes by increasing UTI risk, prolonging hospitalization, and escalating healthcare costs, decision for urinary catheterization should be individualized, based on one’s risk factors [58].

Induction and maintenance of anesthesia should rely on short-acting agents, such as remifentanil, sugammadex, or desflurane, to allow rapid emergence and recovery of airway reflexes. Administration of short-acting, minimally lipophilic anesthetic agents is advantageous, in combination with multimodal analgesic strategies aimed at minimizing perioperative opioid requirements [59]. Thoracic epidural analgesia (TEA) is recommended for open bariatric procedures, ensuring sufficient pain relief while facilitating early mobilization and rapid restoration of gastrointestinal function in patients with severe obesity, particularly those with OSA symptoms, but is not routinely indicated for laparoscopic surgery [60]. In case of contraindication to epidural analgesia or elevated risk of postoperative renal failure or coagulopathy, bilateral transversus abdominis plane blocks or other regional techniques should be considered as alternatives. Whenever feasible, minimally invasive surgical approaches should be preferred. Intraoperative oxygen supplementation with a fraction of inspired oxygen exceeding 50% is recommended [61,62]. Of equal importance, implementation of goal-directed fluid administration protocol using validated monitoring devices should be applied to achieve hemodynamic optimization. This approach helps avoid intraoperative fluid overload in bariatric patients and may contribute to improved outcomes, including a lower incidence of PONV and shorter length of hospital stay [63]. If unavailable, a restrictive fluid strategy based on ideal body weight should be adopted.

Intraoperative hypothermia has been associated with higher rates of seroma formation, increased intraoperative blood loss, and need for transfusion, highlighting the importance of maintaining normothermia during MBS operation [64]. Accordingly, perioperative core temperature must be monitored continuously, and normothermia actively maintained using patient prewarming, maintaining an elevated operating room temperature, and routine therapy with warmed intravenous fluids, and warming blankets. PONV occur in approximately 20–70% of patients following MBS, with incidence varying according to surgical technique, anesthetic management, and individual patient factors. The etiology of PONV in this population is multifactorial, encompassing neurohormonal, inflammatory, mechanical, and pharmacological pathways [65]. POVN prophylaxis should be decided according to the patient’s Apfel risk score, with multimodal antiemetic therapy employed in high-risk individuals.

Beyond perioperative care principles, meticulous attention to key technical aspects of the surgical procedure itself remains fundamental to optimizing outcomes after MBS. Although reinforcement of staple lines during sleeve gastrectomy is widely practiced, there is currently no consensus regarding its necessity. Available evidence indicates that routine use of stapler sleeves or biological glues does not confer additional benefit over meticulous stapling technique alone and does not significantly reduce the risk of staple-line dehiscence [66]. During vertical sleeve gastrectomy, calibration with appropriate bougies or probes is mandatory to ensure standardization of the gastric remnant. The diameter of the calibration bougie is a key technical variable in sleeve gastrectomy, with significant effect on postoperative weight reduction and complication profiles. Bougies in the mid-range of approximately 36–40 Fr appear to provide an acceptable compromise between efficacy and safety in many cases, but final selection is based on individual patient factors, surgeon preference, and institutional practice [67]. Last but not least, nasogastric tubes should be used only intraoperatively for gastric decompression and removed before completion of the procedure, and routine placement of abdominal drains should be avoided. Evidence from large cohort analyses of patients undergoing Roux-en-Y gastric bypass revealed that elimination of postoperative nasogastric decompression did not increase anastomotic dehisence or total complication rates, supporting the avoidance of systematic nasogastric tube placement after bariatric procedures [68].

#### 3.2.3. Immediate Postoperative Phase

The immediate postoperative period is mainly coordinated by the anesthetist and nursing personnel and aims at maintaining patient’s physiological stability and homeostasis while facilitating early functional recovery. Core temperature should be monitored at regular intervals and normothermia actively maintained, as postoperative hypothermia is associated with increased cardiopulmonary complications, infections, risk of blood transfusions, and length of hospital stay [69,70]. Despite the fact that the great majority of MBS are performed laparoscopically, some patients present with pre-existing factors that predispose them to inadequate control of acute postoperative pain, including chronic pain syndromes, long-term opioid use, psychiatric conditions, or a history of poorly managed pain during previous surgical procedures [71]. Pain management should be based on active, preventive multimodal analgesia, with restriction of opioid use in order to minimize adverse effects and to reach a target visual analogue scale (VAS) score of less than 3 [72].

Initiation of early oral intake is recommended approximately 6 h after surgery in the absence of nausea or vomiting, beginning with small volumes of clear fluids. Early mobilization is of the utmost importance during this phase, with patients assisted to sit up in bed within 3 h postoperatively and to begin ambulation within 6 h, taking into account nighttime rest. A recent randomized controlled trial demonstrated that a structured, graded ambulation protocol initiated in the post-anesthesia care unit shortly after laparoscopic sleeve gastrectomy significantly lowered the time to first flatus by 5.4 h and improved quality of recovery scores on postoperative days 0–2 without increasing adverse events [73]. These findings suggest the feasibility of the integration of standardized ultra-early mobilization protocols into ERAS pathways to mitigate postoperative gastrointestinal dysfunction after MBS.

Reduction in thrombotic risk is also a major concern during the immediate postoperative phase, proposing pharmacological thromboprophylaxis with low-molecular-weight heparin to be resumed approximately 12 h after surgery, in addition to ongoing mechanical measures [74]. Apfel risk score remains a gold standard for assessment of PONV risk and antiemetic prophylaxis must be administered accordingly, with multimodal therapy used in high-risk individuals [75]. Given the high incidence of obesity among patients with OSA attending outpatient clinics and the substantial proportion of these individuals awaiting bariatric surgery—of whom up to 40% meet criteria for severe disease—the early reinstatement of continuous positive airway pressure therapy after MBS in diagnosed cases is essential to mitigate the heightened risk of postoperative respiratory complications [76].

### 3.3. Postoperative Day 1

Postoperative day (POD) 1 represents a pivotal stage in the enhanced recovery pathway following MBS, constituting the phase in which the transition from passive recovery to active patient participation is formally established. Close collaboration between surgeons and nursing staff is necessary to promote functional independence while ensuring patient safety. Early feeding is initiated using a liquid hypocaloric diet, advanced progressively according to individual tolerance, as early feeding promotes gastrointestinal motility and reduces postoperative ileus rates and shortens length of hospitalization without increasing the risk of anastomotic or staple-line complications in bariatric patients [77]. Consequently, intravenous fluid administration should be discontinued once adequate oral intake is achieved. Early active mobilization is a cornerstone of recovery on POD 1. Prolonged intravenous fluid administration is associated with delayed gastrointestinal recovery and fluid overload, which may contribute to postoperative nausea, tissue edema, and impaired wound healing. Patients are encouraged to ambulate frequently along the wards, as early walking stimulates gastrointestinal motility, enhances pulmonary ventilation, improves insulin sensitivity, and reduces the risk of venous thromboembolism events [78]. 

Perioperative micronutrient supplementation should follow standardized bariatric nutrition guidelines and be individualized according to procedure type, nutritional status, and laboratory findings [79]. Standard multivitamin supplementation combined with targeted correction of documented deficiencies is generally recommended. Particular attention should be given to iron, vitamin B12, vitamin D, calcium, and protein intake, especially in patients undergoing malabsorptive or revisional procedures [80].

A strategy of opioid-sparing multimodal analgesia should be followed in POD 1, to achieve a VAS score of less than 3 [81]. What is more, early withdrawal of urinary catheter is proposed, if the patient is has ambulated sufficiently and is in place, as it is linked with decreased delayed discharges and length of hospital stay [82]. Current evidence indicates that routine placement of abdominal drains offers limited benefit and should therefore be reserved only for carefully selected high-risk patients [83]. In this way, if abdominal drains were placed intraoperatively, their necessity should be reassessed, with prompt removal encouraged when output and clinical findings permit, in the setting of enhanced recovery protocols.

An additional consideration is the continuation of pharmacological thromboprophylaxis, in combination with mechanical measures, given the persistently elevated risk of venous thromboembolic events in bariatric patients during the early postoperative period 1 [84]. In parallel, respiratory physiotherapy should be instituted, incorporating incentive spirometry and deep-breathing exercises performed multiple times daily. These interventions enhance lung expansion, reduce atelectasis, and decrease the incidence of postoperative pulmonary complications, particularly in patients with obesity or OSA [85].

Together, these measures implemented on postoperative day 1 aim to consolidate early recovery and prepare the patient for timely discharge.

### 3.4. Postoperative Day 2

From POD 2 and after in the surgical ward, the emphasis of patient care shifts toward consolidation of functional recovery and preparation for safe discharge. This phase is coordinated collaboratively mainly by the surgical and nursing teams, with the additional contribution of physiotherapists. The patient progresses to a liquid hypocaloric complete diet per os or, when appropriate, a hypocaloric, high-protein nutritional regimen to ensure adequate protein intake while maintaining gastrointestinal tolerance [86].

Although intraoperative drain placement remains common in complex or high-risk bariatric procedures, accumulating evidence indicates that its routine use is associated with increased length of hospital stay and drain-related surgical site infection (DRSSI) rate as well as higher postoperative morbidity [87]. If abdominal drains were placed intraoperatively, their ongoing necessity should be reassessed on the ward on POD 2 and removal encouraged when clinically appropriate, as routine prolonged drainage offers limited benefit after bariatric surgery. In patients managed with thoracic epidural analgesia, catheter removal should be performed on the ward after verification of normal coagulation parameters and confirmation that the catheter has been completely retrieved. 

Postoperative progression should ideally be guided by functional recovery milestones rather than predefined length-of-stay targets. Suggested criteria for stepwise progression include effective pain control with oral analgesia, early mobilization with independent ambulation or minimal assistance, tolerance of oral fluid and protein intake, stable vital signs, and absence of early postoperative complications such as bleeding, uncontrolled nausea or vomiting, or respiratory compromise [88].

In closing, daily discharge readiness should be assessed by the ward team. Discharge may be considered when there are no complications requiring in-hospital management, vital signs are stable with absence of fever, tachycardia, or tachypnoea, pain is well-tolerated with oral analgesics, and the patient is independently ambulated and tolerates oral intake without adverse effects [89]. Hospital discharge readiness should be assessed using a combination of clinical stability and functional recovery indicators [90]. Suggested discharge criteria include adequate pain control with oral medication, ability to tolerate oral hydration and nutritional supplementation, independent mobilization or return to baseline functional mobility, stable hemodynamic and respiratory parameters, and patient understanding of postoperative instructions, including warning signs and follow-up planning [91,92]. These criteria should be applied within individualized clinical judgment and institutional protocols.

### 3.5. Discharge

At the time of discharge, the surgeon and nurse teams must ensure that patients have received clear, individualized, oral and written instructions regarding oral intake progression, wound management, physical activity, thromboprophylaxis, and post-discharge healthcare support. For the first 1–2 weeks, a low-calorie, blended or puréed diet or a complete hypocaloric high-protein oral nutritional regimen is recommended. This should be followed by a transition to a semi-solid diet after approximately two weeks, with gradual progression to a regular solid diet being generally recommended after 1–2 months, according to tolerance and nutritional status of the patient [93]. Apart from a structured dietary program, a standardized postoperative exercise program should be prescribed, combining aerobic activity with muscle-strengthening exercises [94]. This program is typically initiated one month postoperatively at moderate intensity and participants should progress gradually to higher intensities, tailored to one’s functional capacity and clinical course.

Long-term nutritional monitoring represents a critical component of postoperative bariatric care [95]. Laboratory monitoring is typically recommended at regular intervals (e.g., 3, 6, and 12 months postoperatively and annually thereafter), with adjustment based on clinical status and surgical procedure. Longitudinal monitoring should focus on micronutrient status, protein intake adequacy, bone health, and metabolic adaptation, supported by multidisciplinary follow-up including bariatric specialists, dietitians, and primary care physicians [96]. Last but not least, patient education and adherence to long-term supplementation remain essential for prevention of late nutritional complications.

Patients must be provided with clear wound care instructions, including daily inspection and hygiene, with scheduled removal of sutures or skin staples in accordance with institutional protocols. It should also be noted that patients should be educated to be alert for early signs of wound infection and to seek prompt medical attention upon concern [97]. Venous thromboembolism following MBS is associated with substantial morbidity and mortality, and extended chemoprophylaxis beyond hospital discharge should be prescribed in selected high-risk patients. In particular, individuals with an estimated 30-day VTE risk of at least 0.4% appear to derive measurable benefit from prolonged prophylaxis, supporting a risk-stratified approach to post-discharge anticoagulation [98]. Extended pharmacological thromboprophylaxis is recommended for the first 3–4 weeks following surgery in order to mitigate the persistently elevated risk of venous thromboembolism in bariatric patients. 

In closing, it is also worth emphasizing that continuity of patient care must be ensured through early post-discharge telephone follow-up and coordination of home support with primary care services. In this way, early identification of complications and reinforcing adherence to dietary and lifestyle recommendations are ensured. 

## 4. Discussion

The rapidly expanding global obesity epidemic constitutes one of the most severe public health burdens of modern times, driven by an increasingly obesogenic environment characterized by wide offer of inexpensive energy-dense foods, sedentary technologies, urban infrastructures that discourage physical activity, and dietary patterns that favor refined carbohydrates and added sugars [99]. Forecasting models predict a dramatic escalation in the obesity burden, assessing that up to 42–51% of the population may be obese by 2030, with severe obesity rising to approximately 11%, trends that are expected to markedly intensify the economic and clinical strain on healthcare systems.

Metabolic–bariatric surgery (MBS) is a safe intervention with the most consistent evidence among available obesity treatments for achieving sustained and prolonged weight loss and improving obesity-related comorbidities; nevertheless, despite its proven efficacy, it remains substantially underutilized worldwide [100]. MBS is performed in a uniquely vulnerable patient population characterized by extreme physiological and metabolic challenges. Severe obesity is associated with impaired respiratory mechanics, reduced functional residual capacity, insulin resistance, systemic inflammation, endothelial dysfunction, and a prothrombotic state, all of which amplify perioperative risk [101]. In addition, high rates of OSA, sarcopenic obesity, micronutrient deficiencies, and altered pharmacokinetics complicate anesthetic management and postoperative recovery [102]. These pathophysiological burdens contribute to delayed mobilization, increased postoperative gastrointestinal dysfunction, higher pulmonary and thromboembolic complication risk, and significant variability in pain control, rendering enhanced recovery in bariatric patients substantially more challenging than in most other elective surgical populations.

In recent years, Enhanced Recovery After Surgery (ERAS) pathways have become a cornerstone in modern bariatric practice, offering a structured, multimodal framework aimed at reducing surgical stress, accelerating functional recovery, and reducing postoperative morbidity and length of stay [103,104]. Recent randomized trials and meta-analyses illustrated that ERAS protocols in MBS lead to lower rates of PONV, earlier mobilization, shorter intensive care and overall hospital stay periods, and improved quality of life during recovery period, particularly in cases of compliance with a large number of protocol components [3,105]. However, the application of ERAS in MBS operations encounters several barriers, including the low or very low quality of evidence for many recommendations, the necessity to extrapolate data from non-bariatric surgical populations, and difficulties in standardizing complex behavioral interventions such as prehabilitation, nutritional conditioning, and carbohydrate loading in patients with obesity and diabetes [106]. In addition, challenges arising from heterogeneity in institutional resources and limited multidisciplinary integration continue to pose obstacles to full protocol adherence, highlighting the persistent gap between theoretical guideline recommendations and real-world implementation [107,108].

Despite strong evidence supporting ERAS concepts across several surgical fields, the translation of these principles into bariatric practice remains heterogeneous, with substantial variability between institutions and countries [104]. The present EUPEMEN bariatric protocol seeks to address this gap by suggesting a structured, stage-based, multidisciplinary framework addressing the requirements of bariatric patients, building on the methodological experience and collaborative efforts of five academic specialists with a clinical background dedicated in perioperative care from university healthcare settings across four European countries developing the EUPEMEN project.

A main strength of the proposed protocol is its emphasis on the pre-admission phase, which is frequently underutilized in MBS. Severe obesity is associated with insulin resistance, chronic inflammation, sarcopenia, micronutrient deficiencies, and a high prevalence of obstructive sleep apnea, all of which negatively influence perioperative outcomes [109]. The protocol suggests a series of systematic metabolic, nutritional, respiratory, and cardiovascular optimization measures prior to surgery, integrating dietary prehabilitation, correction of nutrient deficiencies, structured physical conditioning, and cardiopulmonary risk assessment. This approach is in accordance with evidence from other EUPEMEN modules demonstrating that early, multidisciplinary engagement improves adherence, reduces perioperative stress, and enhances functional recovery after surgery [19].

During the intraoperative phase, the protocol establishes the importance of standardized safety measures, including routine use of the WHO Surgical Safety Checklist, goal-directed fluid therapy, opioid-sparing anesthesia, normothermia maintenance, and minimization of invasive vascular catheters and tubes. These proposals are in accordance with the broader EUPEMEN philosophy that avoiding unnecessary devices, ensuring physiological stability, and favoring minimally invasive approaches are fundamental to reducing perioperative stress and postoperative morbidity and accelerating recovery. 

Finally, the postoperative phase emphasizes on timely ambulation, early oral feeding, multimodal analgesia, thromboembolic event prevention, and prompt removal of lines and drains. These interventions aim to counteract postoperative gastrointestinal dysfunction, pulmonary complications, and venous thromboembolism, which rank among the main causes of morbidity after MBS. Importantly, a recent randomized study demonstrated that initiating graded ambulation already in the post-anesthesia care unit significantly shortened the time to first flatus and improved quality of recovery without increasing complications, revealing the potential value of extremely early mobilization within ERAS pathways [73]. Last but not least, one should mention that the transition from hospital to home after MBS constitutes a particularly vulnerable phase of recovery, underscoring the importance of close coordination between the surgeons, nurses, and primary care providers to ensure continuity of follow-up and prompt management of potential complications. The EUPEMEN pathway addresses this need by advocating for a systematic discharge process that incorporates individualized nutritional advice, wound care education, and prearranged follow-up to facilitate early identification and treatment of delayed complications. Reinforcing patient and caregiver comprehension through written resources and remote communication tools further supports sustained adherence to postoperative recovery objectives after hospital discharge.

Another important input of the EUPEMEN protocol is its focus to implementation science. It should be taken into consideration that the primary difficulty to ERAS adoption is not scarcity of evidence but resistance to change, organizational fragmentation, and limited multidisciplinary coordination [110]. The EUPEMEN initiative, by assigning clear and discrete responsibilities to surgeons, anesthetists, nurses, dietitians, and physiotherapists at each perioperative stage, and by incorporating the pathway within a standardized educational and auditing model, the EUPEMEN protocol aims to overcome these obstacles and facilitate reproducible real-world implementation of enhanced recovery principles. In addition, beyond patient-centered benefits, the EUPEMEN protocol also improves workflow efficiency and supports clinicians through structured perioperative information flow, facilitating timely and data-driven perioperative interventions.

However, the EUPEMEN collaborative has some inherent limitations that should be mentioned. The protocol is based on expert consensus and narrative literature review rather than formal meta-analysis. Moreover, the clinical impact of the pathway has not yet been prospectively validated. The scarcity of systematic outcome data significantly limits the ability to determine the long-term effects of the protocol on patient prognosis, economic burden, and overall effectiveness beyond the early postoperative period. In addition, consistent application and implementation of the pathway presupposes the availability of resources, including specialized perioperative monitoring tools and well-coordinated multidisciplinary teams, which may not be feasible in resource-limited settings. Variability in adherence across institutions also represents a major obstacle, as differences in organizational culture, infrastructure, and professional practice can influence the consistency of protocol application. Finally, the EUPEMEN initiative has been developed primarily within a European healthcare framework, which may restrict its transferability to countries with different healthcare structures, patient characteristics, and disease profiles, highlighting the need for contextual adaptation in non-European settings.

## 5. Future Research Directions

Prospectively, the EUPEMEN project intends to conduct prospective, multicenter trials to evaluate the clinical significance of the bariatric protocol on key outcomes such as length of hospital stay, postoperative gastrointestinal function, venous thromboembolism, readmission rates, and overall complication trends. These initiatives will use pre-standardized data record sheets and predefined outcome measures to enable reliable comparisons between centers. Results will be used as a basis for meaningful refinement of the pathway and development of evidence-based principles for perioperative care in MBS. In the same time, the network seeks to broaden its educational activities, strengthen its digital auditing infrastructure, and promote multidisciplinary workshops to improve protocol adherence and interprofessional collaboration. Last but not least, future evaluations will incorporate patient-reported outcome measures, including quality of life, symptom severity, and satisfaction with care provided, ensuring that the protocol evolves in a scientifically robust and patient-centered manner.

To facilitate future validation and inter-institutional comparison, the following core outcome set is proposed for studies evaluating the EUPEMEN bariatric protocol (Table 1).

## 6. Conclusions

To conclude, the EUPEMEN bariatric protocol offers a comprehensive, multidisciplinary, and pragmatic framework for perioperative care in MBS. By translating established ERAS principles into a bariatric-specific, phase-oriented pathway, it aims to limit variability in everyday practice, enhance patient recovery, and assist healthcare professionals in offering consistent, high-quality care across diverse healthcare environments. However, further prospective multicenter studies are necessary to validate its clinical effectiveness and cost–benefit value.

## Figures and Tables

**Table 1:** Suggested outcome measures for future evaluation of the EUPEMEN bariatric protocol.

Outcome Domain	Outcome Measure	Suggested Definition	Suggested Timepoint
Healthcare Utilization	Length of hospital stay	Days from surgery to discharge	Index hospitalization
Healthcare Utilization	ICU admission	Need for postoperative ICU monitoring	During index admission
Safety	Overall postoperative complications	Complications graded by Clavien–Dindo classification	30 days
Safety	Major complications	Clavien–Dindo grade ≥ III	30 days
Safety	Reoperation rate	Return to OR for complication management	30 days
Safety	Readmission	Unplanned hospital readmission	30 days
Recovery	Time to mobilization	Hours to first ambulation	Postoperative day 0–1
Recovery	Time to oral intake	Time to tolerance of fluids and protein supplementation	Postoperative day 0–1
Recovery	Pain control	Numeric Rating Scale (NRS) ≤ 4 with oral analgesia	Discharge
Patient-Centered	Postoperative nausea/vomiting	Need for rescue antiemetics or vomiting episodes	24–48 h
Patient-Centered	Patient satisfaction	Validated patient-reported outcome tools	30 days
Metabolic	Early weight change	% total body weight loss	30–90 days
Long-Term	Sustained weight loss	% total weight loss or % excess weight loss	12 months

## Data Availability

All data generated or analyzed during this study are included in this published article and its Supplementary Materials.

## Supplementary Materials

The following supporting information can be downloaded at: https://www.mdpi.com/article/10.3390/jcm15051706/s1, The full EUPEMEN Protocol for Bariatric Surgery, including methodological details, is available as Supplementary Material.

## Abbreviations

The following abbreviations are used in this manuscript:

ASA	American Society of Anesthesiologists
BMI	Body Mass Index
CPAP	Continuous Positive Airway Pressure
DRSSI	Drain-Related Surgical Site Infection
ERAS	Enhanced Recovery After Surgery
EUPEMEN	EUropean PErioperative MEdical Networking
FiO_2_	Fraction of Inspired Oxygen
GRADE	Grading of Recommendations, Assessment, Development and Evaluation
ICU	Intensive Care Unit
LMWH	Low Molecular Weight Heparin
LOS	Length of Stay
LSG	Laparoscopic Sleeve Gastrectomy
MBSAQIP	Metabolic and Bariatric Surgery Accreditation and Quality Improvement Program
MBS	Metabolic and Bariatric Surgery
OSA	Obstructive Sleep Apnea
PACU	Post-Anesthesia Care Unit
POD	Postoperative Day
PONV	Postoperative Nausea and Vomiting
POUR	Postoperative Urinary Retention
PRISMA	Preferred Reporting Items for Systematic Reviews and Meta-Analyses
QoR-15	Quality of Recovery-15 Questionnaire
RCT	Randomized Controlled Trial
RICA	Recovery Intensification for Optimal Care in Adult Surgery
STOP-BANG	Snoring, Tiredness, Observed Apnea, Blood Pressure, BMI, Age, Neck Circumference, Gender Questionnaire
TAP block	Transversus Abdominis Plane Block
TEA	Thoracic Epidural Analgesia
VAS	Visual Analogue Scale
VTE	Venous Thromboembolism
WHO SSC	World Health Organization Surgical Safety Checklist
