# Supplementary material for: A Phase-Based, Multidisciplinary Enhanced Recovery Pathway for Bariatric Procedures: The EUropean PErioperative MEdical Networking (EUPEMEN) Collaborative for Obesity Surgery
Source: J Clin Med. 2026 Feb 24;15(5):1706. doi: 10.3390/jcm15051706 (PMC12985827; doi:10.3390/jcm15051706)
Supplement: Supplementary file 1 [file jcm-15-01706-s001.zip › jcm-4130880-supplementary.pdf]

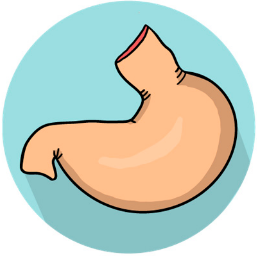

EUPEMEN PROTOCOL

BARIATRIC SURGERY

1	Before Admission<br>Anaesthetist, Surgeon, Nurse, Nutritionist
1.1	**Preoperative counselling**<br>The patient should be fully informed on procedure and perioperative course both verbally and in writing. Signed informed consent should be obtained.
1.2	**Comprehensive medical assessment**<br>This should include medical history, physical examination, chest X-ray, blood tests (coagulation parameters, biochemical profile, nutritional profile and full blood count) and electrocardiogram.
1.3	**Upper digestive endoscopy**<br>Endoscopic evaluation of the esophagus, stomach and duodenum should be performed as part of the preoperative preparation. This should include investigation for *Helicobacter pylori,* which if found should be eradicated before the surgery.
1.4	**Optimization of chronic diseases**<br>All chronic diseases should be optimised before surgery. Preoperative spirometry should be performed for patients with restrictive lung disease. Cardiology evaluation is recommended if the cardiovascular risk score is greater than 3. All cases of recent onset or active cardiovascular diseases should be evaluated by a cardiologist.
1.5	**Evaluation of Diabetes Mellitus**<br>Blood glucose and HbA1c levels should be investigated. All cases of poorly controlled or previously undiagnosed diabetes should be referred to primary care or endocrinology before surgery.
1.6	**Screening for sleep apnoea**<br>Screen for sleep apnoea with the STOP-BANG test. Perform polysomnography if score is greater than 3.
1.7	**Nutritional optimisation**<br>Weight loss before surgery using a very-low-calorie diet or commercial products. Assess adjunctive methods for weight loss (drugs, intragastric balloon). Correction of preoperative nutritional deficiencies including calcium, iron, vitamin D and vitamin B12.
1.8	**Abandon tobacco use and reduce alcohol consumption**<br>Use of tobacco should be stopped and alcohol consumption should be reduced as soon as diagnosis is made.
1.9	**Physical exercises**<br>Cardiovascular, respiratory and muscle strengthening exercises tailored to the physical state of the patient.
1.10	**Psychological counselling**<br>Any psychological issues the patient may be facing should be fully addressed.

EUPEMEN European Perioperative Medical Networking
Co-funded by the Erasmus+ Programme of the European Union
Cátedra GERM Medicina Perioperatoria Universidad Zaragoza

1.11	**ASA assessment**<br>As part of the preoperative anaesthesia assessment the ASA score should be obtained.
1.12	**Apfel score**<br>The risk for postoperative nausea and vomiting (PONV) should be assessed with the Apfel score.
2	Perioperative
2.1	Immediate Preoperative<br>(Schedule admission the same day of surgery if possible)<br><br>Anaesthetist, Surgeon, Nurse
2.1.1	**Preoperative fasting**<br>Patients should not be allowed to eat solid food 8 hours before the surgery and drink liquids 2 hours before the surgery.
2.1.2	**Low molecular weight heparin**<br>Low molecular weight heparin should be administered 2-12 hours before surgery (depending on whether neuraxial anaesthesia is to be performed or not).
2.1.3	**Compression stockings**<br>Placement of compression stockings or intermittent pneumatic compression, according to thromboembolic risk.
2.1.4	**Carbohydrate drink**<br>A single carbohydrate drink (12.5% maltodextrins) 400 ml should be given 2 hours prior to anaesthesia if there is no contraindication.
2.1.5	**Avoid anxiolytic premedication**<br>Do not add preoperative anxiolytic premedication.
2.1.6	**Shaving with electric razor**<br>The site where the incision will be performed should be shaved with an electric razor, if necessary.
2.1.7	**Antibiotic prophylaxis**<br>Antibiotic prophylaxis should be given 30-60 min before surgical incision. The choice of antibiotic should be made based on local hospital protocol.
2.1.8	**Prophylactic measures for prevention of gastric regurgitation**<br>For patients with delayed gastric emptying prophylactic measures should be made to prevent regurgitation.
2.2	Intraoperative<br><br>Anaesthetist, Surgeon, Nurse

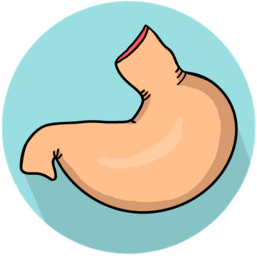

EUPEMEN PROTOCOL

BARIATRIC SURGERY

2.2.1	**WHO Surgical Safety Checklist**<br>The WHO Surgical Safety Checklist should be completed before incision is made.
2.2.2	**Routine intraoperative monitoring**<br>Vital functions, FiO2, anaesthesia depth with, neuromuscular blockade and glycemia should be monitored during the procedure. Non-invasive hemodynamic monitoring is also recommended.
2.2.3	**Avoid arterial catheters**<br>Invasive arterial catheter is not routinely required. Although it should be used for patients with severe cardiorespiratory disorders.
2.2.4	**Avoid central venous catheters**<br>Central venous catheters are not routinely required for minor resections and in the absence of risk factors for postoperative renal failure.
2.2.5	**Avoid routine bladder catheterization**
2.2.6	**Induction and maintenance of anaesthesia**<br>Short-acting agents should be used for induction and maintenance of anaesthesia.
2.2.7	**Oxygenation**<br>Patients should receive oxygen with a FiO2 of more than 50%.
2.2.8	**Fluid therapy**<br>Hemodynamic optimization using goal-guided fluid therapy with validated devices is recommended. If these are not available, restrictive fluid therapy is recommended based on ideal weight.
2.2.9	**Prevention of hypothermia**<br>Temperature should be monitored and normothermia should be maintained by active heating (heated infusions, heated blanket).
2.2.10	**Prophylaxis of postoperative nausea and vomiting**<br>Give antiemetic therapy according to the Apfel score.
2.2.11	**Epidural analgesia**<br>Thoracic epidural analgesia should be used in open surgery. In laparoscopic surgery it is not routinely recommended. Patients with contraindication for epidural analgesia and who have a risk for postoperative renal failure or coagulopathy could benefit from bilateral transverse abdominis plane block or other alternatives to epidural analgesia.
2.2.12	**Minimally invasive surgery**<br>Minimally invasive approaches are preferred and should be used as much as possible.
2.2.13	**Avoid stapler sleeves and biological glues**<br>Staple line reinforcement methods, such as the use of stapler sleeves or biological glues do not reduce dehiscence according to evidence.

EUPEMEN
European Perioperative Medical Networking
Co-funded by the Erasmus+ Programme of the European Union
Cátedra GERM Medicina Perioperatoria
Universidad Zaragoza

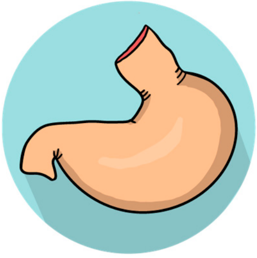

EUPEMEN PROTOCOL

BARIATRIC SURGERY

2.2.14	**Calibrate the vertical (sleeve) gastrectomy**<br>The vertical (sleeve) gastrectomy must be calibrated with probes.
2.2.15	**Avoid nasogastric tubes**<br>Nasogastric tubes are only recommended intraoperatively in order to empty the stomach.
2.2.16	**Avoid abdominal drains**
**2.3**	**Immediate Postoperative**<br><br>Anaesthetist, Nurse
2.3.1	**Maintenance of normothermia**<br>Temperature should be regularly measured and normothermia should be maintained.
2.3.2	**Opioid sparing analgesia**<br>Active or preventive multimodal analgesia should be used. Restrict the use of opioids. Aim for a VAS score of less than 3.
2.3.3	**Early feeding**<br>Beginning of oral fluid intake from 6 hours after the surgery.
2.3.4	**Early mobilisation**<br>Mobilization should begin 3 hours after surgery and should begin with sitting up in bed. Ambulation should begin 6 hours after surgery with respect to night time hours for sleeping.
2.3.5	**Thromboembolic prophylaxis**<br>Low Molecular Weight Heparin should be given 12 hours after surgery.
2.3.6	**Prophylaxis of postoperative nausea and vomiting**<br>Give antiemetic therapy according to the Apfel score.
2.3.7	**Sleep apnoea treatment**<br>In patients with sleep apnoea, reinstate Continuous Positive Airway Pressure as soon as possible.
**3**	**Postoperative Day 1**<br>(Ward)<br><br>Surgeon, Nurse
3.1	**Early feeding**<br>A liquid hypocaloric diet should be started, according to patient tolerance.
3.2	**Early mobilization**<br>Patients should be encouraged to walk.
3.3	**Opioid sparing analgesia**

EUPEMEN
European Perioperative Medical Networking
Co-funded by the Erasmus+ Programme of the European Union
Cátedra GERM Medicina Perioperatoria
Universidad Zaragoza

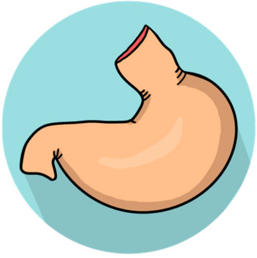

EUPEMEN PROTOCOL

BARIATRIC SURGERY

	Active or preventive multimodal analgesia should be used. Restrict the use of opioids. Aim for a VAS score of less than 3.
3.4	**Stop intravenous fluids**<br>If patients tolerate adequate oral liquid intake, intravenous fluid therapy should be discontinued.
3.5	**Remove urinary catheter**<br>Evaluate withdrawal of urinary catheter if one is in place.
3.6	**Removal of abdominal drains**<br>If a drain has been placed assess its removal.
3.7	**Thromboprophylaxis**
3.8	**Respiratory physiotherapy**
4	Postoperative Day 2 (and following)<br>(Ward)<br><br>Surgeon, Nurse
4.1	**Early feeding**<br>Give patients a liquid hypocaloric complete diet or hypocaloric high-protein complete nutrition.
4.2	**Remove abdominal drains**<br>Evaluate removal of drains if they have been placed.
4.3	**Assess discharge**<br>Consider discharge if there are complications that cannot be managed in an outpatient setting if there is no fever, no tachycardia or tachypnoea, if pain can be controlled with peroral analgesia, the patient is fully ambulated and can tolerate an oral diet.
5	At discharge<br><br>Surgeon, Nurse
5.1	**Diet**<br>A hypocaloric turmix diet or complete hypocaloric high-protein oral nutrition is recommended for the first 1-2 weeks. After 2 weeks semi-solid diet. Solid diet should commence 1-2 months after surgery.
5.2	**Wound care**<br>Daily wound care and removal of stitches or skin staples according to protocol.
5.3	**Exercise**<br>Exercise program that combines aerobic and strength training. Start from 1 month after surgery at moderate intensities progressing to higher intensities.

EUPEMEN
European Perioperative Medical Networking
Co-funded by the Erasmus+ Programme of the European Union
Cátedra GERM Medicina Perioperatoria
Universidad Zaragoza

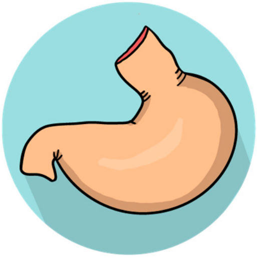

5.4	**Thromboprophylaxis**<br>Thromboprophylaxis is recommended for the first 3-4 weeks post-operatively
5.5	**Continued care**<br>Telephone control after discharge. Home support coordination with primary care
